# Elasticity Anisotropy of *Bombyx mori* Silkworm Silk Fiber by Brillouin Light Spectroscopy

**DOI:** 10.1021/acs.biomac.4c01844

**Published:** 2025-04-01

**Authors:** Alina Aluculesei, Yuanzhong Zhang, Shifeng Huang, Zuyuan Wang, Yu Cang, Younjin Min, George Fytas

**Affiliations:** 1 Institute of Electronic Structure and Laser, FORTH, N. Plastira 100, Heraklion 70013, Greece; 2 Department of Chemical and Environmental Engineering, University of California, Riverside, California 92521, United States; 3 School of Mechanical and Electrical Engineering, University of Electronic Science and Technology of China, Chengdu, Sichuan 611731, PR China; 4 School of Aerospace Engineering and Applied Mechanics, Tongji University, Zhangwu Road 100, Shanghai 200092, China; 5 Material Science and Engineering Program, University of California, Riverside, California 92521, United States; 6 Max Planck Institute for Polymer Research, Ackermannweg 10, Mainz 55128, Germany

## Abstract

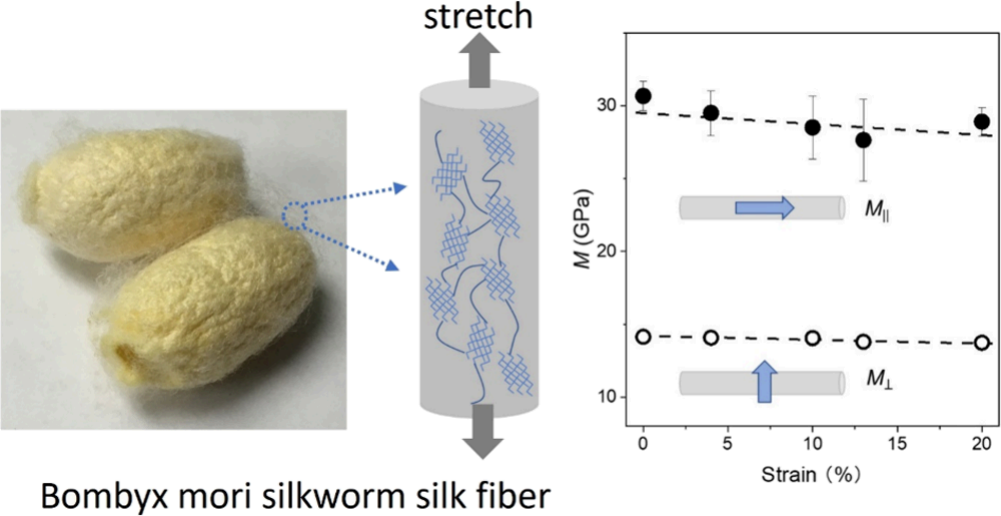

Silkworm silk has long been an important natural protein fiber for textile and medical applications, where its superior mechanical properties play a crucial role. Despite the many studies by conventional stress–strain tests, our understanding of the mechanical properties of silkworm silk remains limited. This work investigates the complete elastic properties of *Bombyx mori* silkworm silk in a noncontact, noninvasive manner by conducting Brillouin light spectroscopy experiments. The analysis of the angle-dependent sound velocities leads to the determination of the full elastic tensor and the engineering mechanical properties of the silkworm silk in natural and stretched states. In the natural state, the axial and lateral Young’s moduli are 23.4 ± 1.0 and 10.4 ± 0.5 GPa, respectively, giving an elastic anisotropy of 2.3. Different from the strain-hardening behavior of the spider silk, the mechanical properties of the silkworm silk exhibit a weak strain-dependence up to the breakage strain (∼20%).

## 1 Introduction

Silk has long captivated scientists and engineers owing to its superior mechanical properties, biocompatibility, and versatility as a natural protein fiber.^1,2^ Both cocoon silk, produced by silkworms such as *Bombyx mori*, and dragline silk from spiders exhibit impressive combinations of strength, extensibility, and toughness that exceed many synthetic polymer fibers.^3^ The hierarchical nanostructure of silk fibroin proteins, consisting of β-sheet nanocrystals embedded in an amorphous matrix, is responsible for these exceptional mechanical characteristics.^4,5^ In spider silk, particularly dragline silk, the protein architecture involves organized molecular structure, where long, aligned protein chains form strong β-sheet layers interspersed with flexible, amorphous regions.^6^ In cocoon silk, the protein architecture typically involves a less-organized architecture with shorter fibroin protein chains and a higher proportion of amorphous regions.^7^ These unique architectures allow silk to dissipate energy effectively through sacrificial bond breaking and molecular alignment under strain.^7,8^

The versatility and exceptional properties of silk have encouraged extensive research into its potential applications across diverse fields. In biomedicine, silk-based materials are being explored for drug delivery systems,^9,10^ tissue engineering scaffolds,^11,12^ and wound dressings,^13,14^ utilizing its biocompatibility and controllable biodegradability.^15^ Surgical applications include sutures, meshes, and bone grafts, while implantology has seen investigations into silk-derived orthopedic, dental, and soft tissue implants.^16,17^ Its optical properties and biocompatibility have led to developments in ophthalmology, with silk-based contact lenses and intraocular implants under study.^18,19^ Bioelectronics represents another frontier, with silk being investigated for flexible electrodes, biosensors, and implantable devices.^20,21^ Beyond medicine, its mechanical properties are being relied on for high-performance textiles, protective gear, and even aerospace components.^22,23^ Environmental applications include water purification membranes and air filtration systems.^24,25^ The food industry is exploring silk for edible coatings and nutritional supplements.^26,27^ Energy storage applications, such as battery separators and supercapacitor electrodes, are also being investigated.^28,29^ Numerous applications of silk underscore the importance of fundamental studies on its structure–property relationships, as exemplified by the current investigation.

Regarding the mechanical properties of silk, a careful comparison between cocoon and spider silk is necessary to elucidate the structure–property relationships that can lead to different mechanical behaviors. For spider dragline silk, researchers have employed a combination of techniques including quasi-static tensile testing,^30,31^ atomic force microscopy-based indentation,^32,33^ Brillouin light scattering,^34−36^ and molecular dynamics simulations^37,38^ to determine the independent elastic constants that define its elastic tensor. These studies revealed the highly anisotropic nature of spider silk, with the longitudinal elastic constant (*C*_33_) being over two times the transverse one (*C*_11_).^36^ Prior studies have also revealed that spider silk typically exhibits a higher tensile strength, extensibility, and toughness than silkworm silk, although they both rely on similar protein building blocks.^39,40^ Conversely, the full elastic tensor for silkworm cocoon silk has not yet been experimentally determined extensively. Most mechanical characterizations in the literature have focused on tensile properties along the fiber axis.^41−43^ Accessing the complete set of elastic constants for cocoon silk, as has been done for spider silk, would enable a more rigorous comparison between these two natural fibers.

The exceptional mechanical properties of silk fibers from *Bombyx mori* silkworms and *Nephila pilipes* spiders stem from differences in their protein structure, folding, composition, and supramolecular assembly.^44,45^ Both silks are primarily composed of fibroin proteins, but their specific amino acid sequences and structural organization can differ, leading to differing mechanical behaviors.^44,45^*Bombyx mori* silk fibroin consists of a “heavy” chain (∼390 kDa) and a “light” chain (∼26 kDa) linked by a disulfide bond, along with a glycoprotein P25.^46−48^ The heavy chain contains highly repetitive (GAGAGS) motifs that form β-sheet crystalline regions, interspersed with less organized domains.^1^ In contrast, *Nephila pilipes* major ampullate (dragline) silk is composed of two main proteins, MaSp1 and MaSp2, with molecular weights around 250–320 kDa.^49,50^ These proteins feature repetitive motifs rich in alanine (forming β-sheet crystals) and glycine-rich regions (contributing to extensibility).^37^ The supramolecular assembly of these proteins differs between the two silks to some extent. *Bombyx mori* silk fibroin forms a hierarchical structure with β-sheet nanocrystals (∼2–5 nm) embedded in a semiamorphous matrix.^51^ These nanocrystals align partially along the fiber axis, creating a structure with moderate anisotropy.^52,53^*Nephila pilipes* dragline silk features highly aligned β-sheet nanocrystals (with side lengths ∼2–4 nm), where the nanocrystals are interconnected by a network of less-ordered domains rich in β-turns and 31-helices.^13^ Furthermore, the arrangement of secondary structures within the proteins contributes to their distinct mechanical properties. *Bombyx mori* silk relies heavily on the hydrogen bonding within and between β-sheets, with some contributions from hydrophobic interactions.^54^*Nephila pilipes* dragline silk, in addition to these forces, benefits from a higher proportion of proline residues in MaSp2, which introduce kinks in the protein backbone and contribute to elasticity.^55,56^ Moreover, the presence of tyrosine in spider silk allows for π-π stacking interactions,^57,58^ which can partly be responsible for its enhanced tensile strength and toughness.

Despite extensive research, knowledge gaps remain in fully characterizing the anisotropic elastic properties of silk and their evolution under applied strain. For instance, while the axial properties of silk fibers are well studied, their transverse mechanical behavior and structure–property relationships are hardly understood but critically important for composite applications. Furthermore, deeper investigations are necessary to understand the roles of the different hierarchical structural elements --- from individual protein domains to nanofibrils and microfibrils --- in determining the bulk mechanical properties. Structure characterization techniques, such as polarized Raman spectroscopy and synchrotron X-ray scattering, and all-optical, nondestructive Brillouin light spectroscopy for complete determination of the elasticity anisotropy can provide new insights into the structure–property-processing relationships in silk materials. Such fundamental understanding is crucial for developing biomimetic spinning processes and designed protein sequences that can achieve or even exceed the mechanical performance of natural silks for next-generation sustainable high-performance materials.^59,60^ This study aims to compare the anisotropic elasticity of silkworm silk fibers with the isotropic elasticity of the silkworm silk fibroin in the context of their hierarchical structure, providing a comprehensive analysis of the structure–property relationships in these remarkable natural materials.

## 2 Materials and Methods

### 2.1 Materials

Cocoons from the *Bombyx mori* silkworm species were acquired from a commercial supplier in the Pacific Northwest, USA. The chemical agents employed for silk fibroin fiber extraction included sodium carbonate (Na_2_CO_3_, purity >99%), lithium bromide (LiBr, purity >98%), and silver nitrate (AgNO_3_, purity >98%), all procured from Sigma-Aldrich (Saint Louis, MO) and utilized without further purification. Throughout the experimental procedures, ultrapure water with a minimum resistivity of 18 MΩ·cm was used, unless otherwise specified.

### 2.2 Silk Fibroin Extraction and Solution Preparation

The extraction of silk fibroin protein from *Bombyx mori* cocoons followed a modified version of the established protocols.^61−63^ In brief, the process involved degumming 5 g of cocoon material in 2 L of a dilute Na_2_CO_3_ solution (0.02 M) at boiling temperature for 30 min to eliminate the sericin coating. After removing excess sodium carbonate and complete drying, the degummed silk was solubilized in a concentrated LiBr solution (9.3 M) at an elevated temperature (60 °C) for approximately 4 h, using a ratio of 1 g silk to 4 mL solution. The resulted mixture was then subjected to dialysis using semipermeable membranes with a 15 kDa molecular weight cutoff. This dialysis process, conducted over 48 h with six medium changes, served to eliminate residual LiBr from the silk fibroin solution. The dialysis end point was verified using a AgNO_3_ test solution (0.1 M), relying on the low solubility of AgBr to confirm the absence of Br^–^ ions, as indicated by the lack of precipitate formation.

### 2.3 Brillouin Light Spectroscopy (BLS)

BLS is a noninvasive technique based on the inelastic scattering of light by thermally excited hypersonic (GHz) phonons. A green laser (λ = 532 nm in air) with an input power of around 5 mW and a focal spot size of 5.0 μm provided the incident light with a wave vector **k**_i_. The scattered light with a wave vector **k**_s_ was frequency resolved by a six-path tandem Fabry–Perot interferometer (JRS Instruments). For isotropic media, the BLS spectrum at the probed phonon wave vector, **q** = **k**_s_ – **k**_i_, consists of a doublet at ω_B_ = ± *cq*, where *c* is the speed of sound. Depending on the polarization of the incident and scattered light selected to be either vertical (V) or horizontal (H) to the scattering plane defined by **k**_i_ and **k**_s_, a longitudinal (L) or transverse (T) phone mode is obtained in the VV or VH spectrum. The simple situation is changed in the case of mechanically anisotropic media, where the observed phonons depend on the direction of **q** with respect to the symmetry axis. We conducted measurements in transmission and reflection geometries (Figure S1) to access phonons propagating in different directions. The measurements were conducted on both single and bundle of silkworm fibers. The frequency shifts ω_B_ from the BLS measurements were used to calculate the direction-dependent sound velocities, which were subsequently utilized to calculate the stiffness elastic constants and mechanical moduli. More details about the experiments and data analysis are available in Sections S2–S6.

## 3 Results and Discussion

### 3.1 Isotropic Silk Fibroin Thin Film

**Figure 1:**
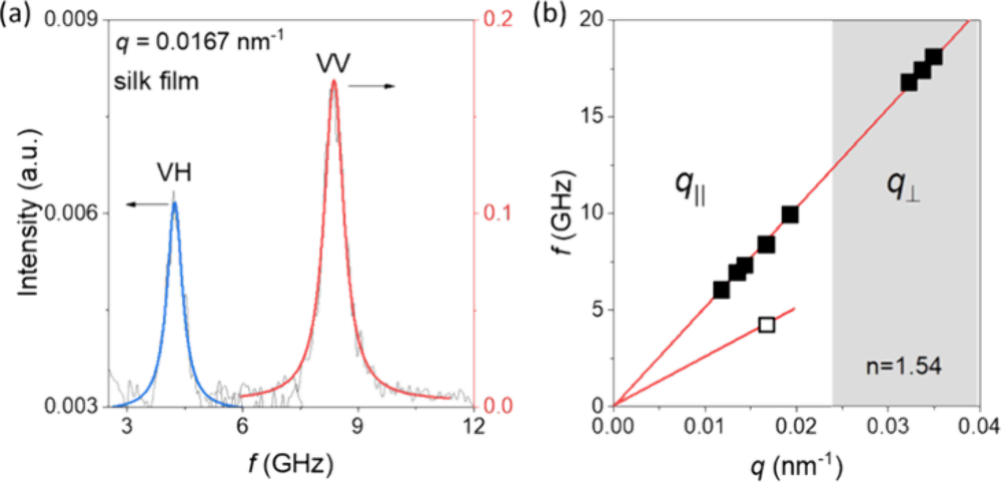
(a) Polarized (VV) and depolarized (VH) spectra (anti-Stokes side) of the silk fibroin film recorded in the 90° transmission geometry at a phonon wave vector, *q*_//_ = 0.0167 nm^–1^. Note the different intensity scales of the VH and VV spectra. (b) Phonon dispersions of the silk fibroin film along the *q*_//_ and *q*_⊥_ directions. The linear acoustic dispersion is obtained with the refractive index *n* = 1.54 entering the expression of the *q*_⊥_, which is normal to the film surface in the shaded region.

Polarized (VV) and depolarized (VH) BLS spectra were recorded at different scattering angles θ = 2β between **k**_i_ and **k**_s_ in the transmission and reflection geometries. For the transmission geometry, the phonon wave vector **q**_**//**_ is directed parallel to the film surface with a magnitude *q*_//_ = (4π/λ) sin(θ/2) being independent of the refractive index *n*. In the reflection geometry with β = 90° - θ/2, **q**_⊥_ is directed normal to the film surface with a magnitude *q*_⊥_ = (4π/λ)[(*n*^2^-cos^2^(θ/2)]^1/2^, which depends on *n*. For a silk fibroin film treated with water-MeOH mixture for 60 min, Figure 1a displays the polarized and depolarized spectra (anti-Stokes side) recorded in the transmission scattering geometry at *q*_//_ = 0.0167 nm^–1^ (θ = 90°). As expected for an elastically isotropic material, each spectrum at a given *q*_//_ consists of a single peak, independent of the **q**_**//**_ direction in the film plane. The VH spectrum is significantly weaker than the VV spectrum due to the low optical anisotropy of the material. Figure 1b displays the phonon frequency *f* vs q_//_ and *q*_⊥_ (shaded area) dispersion, which is found to be linear when *n* = 1.54 is used to calculate *q*_⊥_. Based on the longitudinal sound velocity *c*_L_ = 3235 ± 50 m/s and transverse sound velocity *c*_T_ = 1590 ± 25 m/s, obtained from the slopes in Figure 1b, and the density ρ = 1.17 g/cm^3^, the longitudinal modulus M = ρ *c*_L_^2^ and shear modulus *G* = ρ *c*_T_^2^ are calculated to be 12.2 ± 0.4 GPa and 3.0 ± 0.1 GPa, respectively; the Poisson’s ratio *v* = (*x*^2^-2)/(*x*^2^-1), with *x* = (*c*_L_/*c*_T_) ^2^, assumes a value of 0.34, which is typical for amorphous glassy polymers.

For comparison, the amorphous regenerated spider silk film assumes a lower *c*_L_ = 2920 m/s,^35^ whereas for amorphous (Silk I) and thermally annealed semicrystalline (Silk II) silk fibroin films, *c*_L_ = 3085 ± 40 m/s with *v* = 0.33 (*c*_T_ = 1540 m/s).^64^ It seems that the speed of sound in the amorphous films obtained from both spider silk and silk worm fibers assume similar values. This similarity could stem from the comparable local chain dynamics and intermolecular interactions in their amorphous regions, despite the differences in their primary protein structures. Both types of silks contain glycine-rich repetitive motifs in their amorphous domains, which contribute to the chain flexibility and elastic behavior.^65−67^ In spider silk, these are typically (GGX)*_n_* sequences, while in silkworm silk, they are often (GAGAGS)*_n_* repeats.^68,69^ These repetitive sequences, when in an amorphous state, may adopt similar local conformations, such as random coils and loosely structured helical regions. The presence of these similar secondary structures in the amorphous domains could lead to comparable short-range order and thus similar acoustic phonon propagation characteristics.

### 3.2 Anisotropic Elasticity

**Figure 2:**
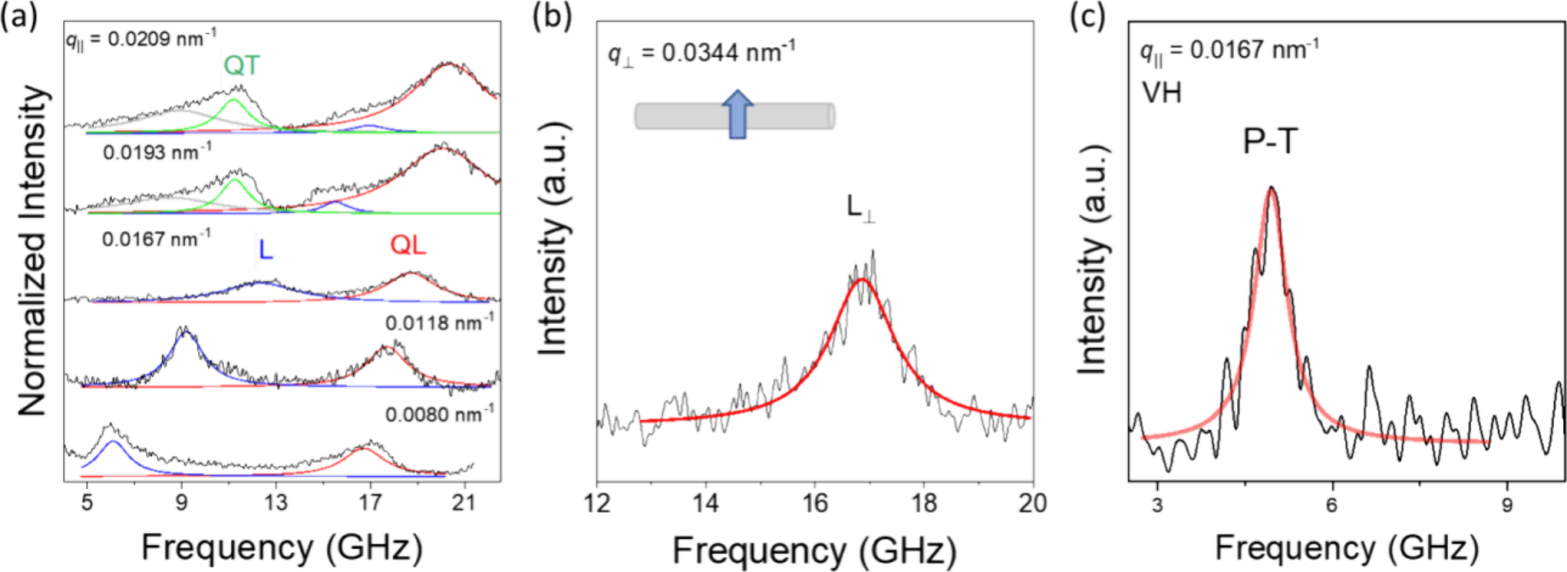
(a) Polarized BLS spectra at selected *q*_//_’s parallel to the silkworm fiber axis. The spectral peaks are represented by Lorentzian line shapes (solid lines). The green, blue, and red lines denote the quasi-transverse (QT, green), longitudinal (L_//_, blue, parallel to the axis), and quasi-longitudinal (QL) phonons, respectively. Note that the acoustic L_//_ becomes weaker at *q*_//_ > 0.0167 nm^–1^. (b) Polarized spectrum recorded in the reflection geometry (θ = 120°, *q*_⊥_ = 0.0344 nm^–1^) due to the longitudinal phonon L_⊥_ normal to the silk fiber axis. (c) Depolarized (VH) spectrum at *q*_//_ = 0.0167 nm^–1^ (θ = 90°) due to the pure transverse (PT) phonon.

The BLS spectra of the silk fiber display more than one phonon at a given *q*_//,_ as shown in the polarized spectrum (the anti-Stokes side) in Figure 2. The low frequency peak (blue Lorentzian fit), termed L_//_, displays a linear dependence with *q*_//_, yielding a high value of *c*_//_ (= 4960 ± 70 m/s along the fiber axis. The intensity of the L_//_ mode strongly decreases with *q*_//_ (>0.0167 nm^–1^), while a second low-frequency peak (QT) emerges. The strong and broad high-frequency peak (QL) is reminiscent of a higher *q* than *q*_//_. It originates from the artificial backscattering (Figure S1) forming an oblique angle α = arc(*n*^–1^sinθ) with the fiber axis, and the mode is assigned as QL at *q*_bs_ = 4π*n*/λ. The intensity of this mode is found to strongly decrease with increasing oblique angle α (between **q** and the fiber axis) (Figure S2).^36,70^ Phonon propagation normal to the fiber axis is probed in the reflection scattering configuration yielding the longitudinal phonon L_⊥_, which is shown in Figure 2b at *q*_⊥_ = 0.0344 nm^–1^ (θ = 120°). The longitudinal sound velocity normal to the fiber axis, *c*_L, ⊥_ (= 3100 ± 60 m/s, Figure 2b), is slightly (∼5%) lower than the isotropic *c*_L_ in the fibroin film. The transverse sound velocity *c*_T_ (= 1860 ± 30 m/s) is obtained from the pure transverse (PT) mode in the depolarized BLS spectrum shown in Figure 2c at *q*_//_ = 0.0167 nm^–1^ (θ = 90°). Note that *c*_T_ along the fiber axis is now larger than the *c*_T_ in the isotropic fibroin film (Figure 1a) yielding an about 60% higher *G* (= 4.7 GPa); for the silkworm fiber, ρ = 1.35 g/cm^3^.

**Figure 3:**
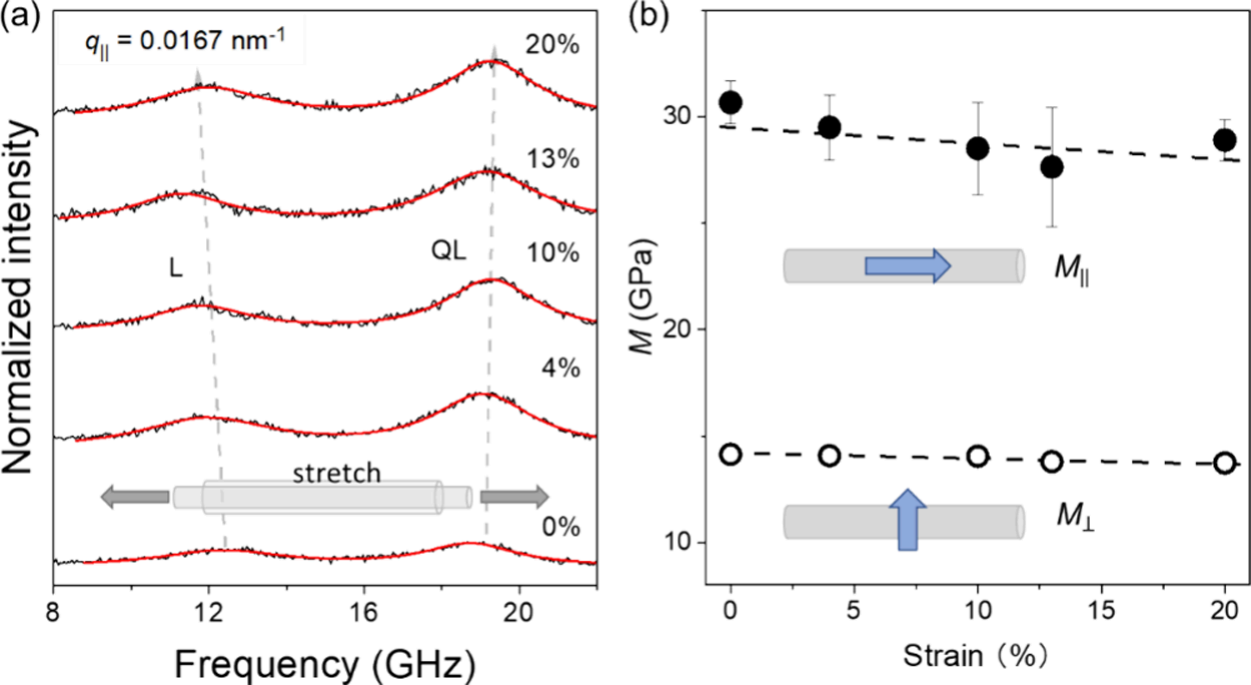
(a) Polarized BLS spectra (anti-Stokes) of silkworm fibers at *q*_//_ = 0.0167 nm^–1^ under different tensile strains (0–20%) along the fiber axis (inset). (b) Longitudinal modulus *M* parallel and normal to the fiber axis (i.e., *M*_||_ and *M*_⊥_) at different strains.

In the case of spider silk, unidirectional stretching leads to material hardening along the fiber due to better aligned semicrystalline chains,^35,36^ whereas a strain weakening, due to β-sheet crystallite fragmentation, was reported for silkworm fiber.^52,71^ To examine this distinct strain effect of the high frequency elasticity, BLS experiments under tensile strains were performed at directions parallel and normal to the fiber axis. Figure 3a shows the polarized BLS spectra at *q*_//_ = 0.0167 nm^–1^ (θ = 90°) along the fiber axis at different strains. The L_//_ shows a softening up to about 15% tensile strain along the fiber axis. *M*_||_ slightly decreases with the strain, whereas *M*_⊥_ (∼14 GPa) along the normal direction is robust to the strain variation (Figure 3b). The somewhat stronger strain-dependence of QL than L_//_ (Figure 3a) is due to the different angles formed by **q**_//_ (α = 0°) and **q**_QL_ (α = 63°) with the fiber axis at θ = 90° (i.e., **q**_QL_ is close to the normal direction). Since the tensile strain mainly affects the mechanical properties along the fiber axis (e.g., chain breakage, which decreases *f*_L//_), *f*_QL_ exhibits a weak strain-dependence.

**Figure 4:**
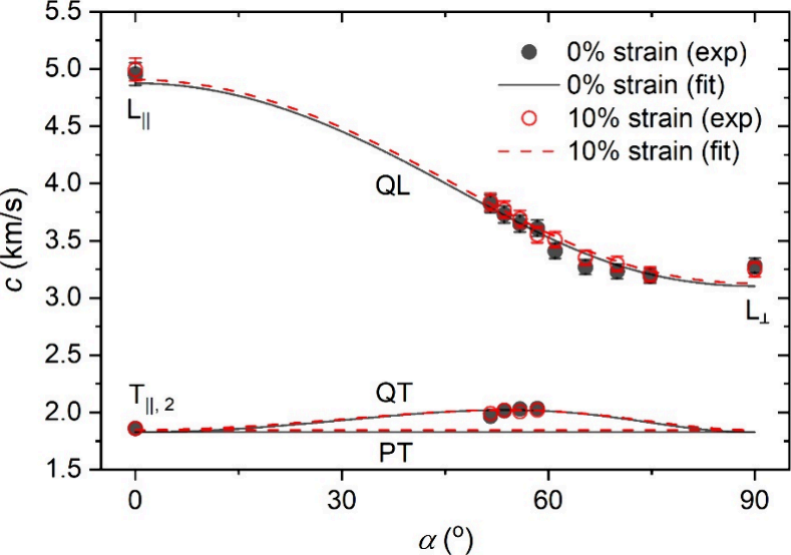
Angle-dependent sound velocities in the native (0% strain) and stretched (10% strain) *Bombyx mori* silk fibers. The lines represent theoretical predictions by eqs S1–S7. The experimental error bars are comparable with or smaller than the symbol size.

Based on the wave vector magnitude *q* and frequency *f* of a phonon mode in the recorded BLS spectrum, the sound velocity of the phonon mode can be calculated as *c* = 2π*f* /*q*. Figure 4 presents the angle-dependent sound velocities of the silkworm silk at 0% and 10% tensile strains, which were measured using VV and VH polarization configurations at transmission and reflection geometries. In general, the probed phonons can be categorized into QL, QT, and PT modes, which become pure modes at α = 0° and 90°. The solid and dashed lines represent least-squares fits of the experimental data according to eqs S1–S7. At 0% strain, as α increases from 0° to 90°, the sound velocity of the QL mode decreases monotonically from 4960 to 3040 m/s, that of the QT mode first increases and then decreases, and that of the PT mode remains constant due to the assumption of *C*_66_ = *C*_44_. The sound velocities at 10% strain nearly overlap with those at 0% strain, indicating a weak strain-dependence of the sound velocities of the silkworm silk.

The determination of the complete elastic constants and the subsequent calculation of the engineering moduli follow the presentation of *c*(α) according to the Christoffel’s equations. Assuming the silkworm silk to be transversely isotropic reduces the number of independent elastic stiffness constants (i.e., *C*_*ij*_ with *i*, *j* ∈ {1, 2, 3, 4, 5, 6 and *i* ≥ *j*)^72,4−6,64^ from 21 to 5. Here, we choose *C*_11_, *C*_33_, *C*_44_, *C*_13_, and *C*_66_ as the independent elastic constants and note that *C*_12_ = *C*_11_ – 2*C*_66_. Since the PT mode is nondetectable except at α = 0°, the assumption of *C*_66_ = *C*_44_ is made,^15^ further reducing the number of independent elastic constants to four (i.e., *C*_11_, *C*_33_, *C*_44_, *C*_13_). The *c*(α) data of the silkworm silk at 0% and 10% strains are analyzed using the method described in the reference^36^ to obtain the elastic stiffness constants, which are further used to calculate the engineering mechanical properties. Table 1 compiles the elastic stiffness constants of the silkworm silk and spider silk at 0% and 10% strains. As the “3” axis aligns with the fiber axis, *C*_33_ takes the largest value, followed by *C*_11_, *C*_13_, *C*_12_, and *C*_44_ = *C*_66_. While the *C*_*ij*_’s of the silkworm silk exhibit a weak strain-dependence (i.e., within the error bar) up to 10% strain, those of the spider silk show a more apparent strain-dependence, especially for *C*_33_, which increases from 26.6 ± 0.8 GPa at 0% strain to 30.3 ± 1.2 GPa at 10% strain.

**Table 1:** Summary of the Elastic Stiffness Constants of the *Bombyx mori* Silk and *Nephila pilipes* Major Ampullate Silk at Two Strains (i.e., 0%, 10%)^a^

Strain	*C*_11_ [GPa]	*C*_13_ [GPa]	*C*_33_ [GPa]	*C*_44_ [GPa]	*C*_66_ [GPa]	*C*_12_ [GPa]
0% (silkworm silk)	13.0 ± 0.3	8.6 ± 0.3	32.1 ± 1.0	4.5 ± 0.1	4.5 ± 0.1	4.0 ± 0.4
10%(silkworm silk)	13.2 ± 0.3	8.9 ± 0.3	32.5 ± 1.0	4.6 ± 0.1	4.6 ± 0.1	4.0 ± 0.4
0% (spider silk)	14.4 ± 0.3	8.0 ± 0.3	26.6 ± 0.8	4.1 ± 0.1	3.1 ± 0.1	8.2 ± 0.4
10% (spider silk)	13.8 ± 0.3	6.9 ± 0.5	30.3 ± 1.2	4.2 ± 0.5	3.9 ± 1.0	5.9 ± 1.9

a Note that *C*_66_ = *C*_44_ is assumed for the *Bombyx mori* silk.

The engineering mechanical properties (including the axial and lateral Young’s moduli, shear moduli, bulk moduli, and Poisson’s ratios) of the silkworm silk and spider silk at 0% and 10% strains are shown in Table 2. At 0% strain, the axial and lateral Young’s moduli are 23.4 ± 1.0 and 10.4 ± 0.5 GPa, respectively, leading to an anisotropy ratio of 2.3. The three characteristic shear moduli *G*_13/23/12_ are 4.5 ± 0.2 GPa, which is higher than typical value (∼3 GPa) for polymer glasses or soft materials. The bulk modulus is 8.5 ± 0.3 GPa. The three characteristic Poisson’s ratios are ν_31/32_ = 0.51 ± 0.02 and ν_12_ = 0.16 ± 0.04. The seemingly unreasonable value of ν_31/32_ greater than 0.5 could stem from the assumption of *C*_66_ = *C*_44_. Since ν_31_ = ν_32_ = *C*_13_/[2(*C*_11_ – *C*_66_)], a smaller *C*_66_ could lead to a ν_31/32_ lower than 0.5. On the other hand, the Poisson’s ratio of anisotropic materials could have no bound,^73^ and previously we also obtained a Poisson’s ratio of 0.51 for an itraconazole molecular glass thin film.^74^ At 10% strain, nearly identical mechanical properties are obtained, consistent with the weakly strain-dependent elastic constants of the silkworm silk in Table 1.

**Table 2:** Summary of the Axial and Lateral Young’s Moduli, Shear Moduli, Bulk Moduli, and Poisson’s Ratios of the *Bombyx Mori* Silk and *Nephila Pilipes* Major Ampullate Silk At Two Strains (i.e., 0%, 10%)^a^

Strain	*E*_||_ [GPa]	*E*_⊥_ [GPa]	*E*_||_/*E*_⊥_	*G*_13/23_ [GPa]	*G*_12_ [GPa]	*K* [GPa]	ν_31/32_	ν_12_
0% (silkworm silk)	23.4 ± 1.0	10.4 ± 0.5	2.3	4.5 ± 0.2	4.5 ± 0.2	8.5 ± 0.3	0.51 ± 0.02	0.16 ± 0.04
10% (silkworm silk)	23.3 ± 1.1	10.5 ± 0.5	2.2	4.6 ± 0.2	4.6 ± 0.2	8.6 ± 0.4	0.52 ± 0.02	0.15 ± 0.04
0% (spider silk)	20.9 ± 0.8	9.2 ± 0.3	2.3	4.1 ± 0.1	3.1 ± 0.1	10.8 ± 0.2	0.35 ± 0.02	0.48 ± 0.03
10% (spider silk)	25.5 ± 1.2	10.7 ± 1.4	2.4	4.2 ± 0.5	3.9 ± 1.0	9.5 ± 0.8	0.35 ± 0.04	0.36 ± 0.16

a Note that *G*_12_ = *G*_23_ holds for the *Bombyx Mori* silk due to the assumption of *C*_66_ = *C*_44_.

As a comparison, the axial Young’s modulus of the spider silk increases from 20.9 ± 0.8 GPa at 0% strain to 25.5 ± 1.2 GPa, exhibiting a clear strain-hardening behavior. Concomitantly, the lateral Young’s modulus increases from 9.2 ± 0.3 to 10.7 ± 1.4 GPa, and the anisotropy ratio from 2.3 to 2.4. The different strain-dependences of the axial and lateral Young’s moduli of the silkworm silk and spider silk could be attributed to their different hierarchical structures.^52,75,76^ Interestingly, the two types of silk have a similar Young’s modulus anisotropy ratio at around 2.3. The shear and bulk moduli of the spider silk are slightly lower and higher than those of the silkworm silk, respectively, whereas the Poisson’s ratios are closer to 0.33 than those of the silkworm silk, which is a typical value for polymers.^77,78^ The isotropic elasticity of the fibroin films is characterized by M = 12.2 ± 0.4 GPa and 3.0 ± 0.1 GPa, which are respectively higher and lower than *C*_11_ and *C*_44_ of the silkworm silk at zero strain (Table 1).

In the case of spider silk fiber, the elastic moduli, listed in Table 2 at two strains, assume similar values with those of the silkworm silk but display a hardening trend under tensile strain.^35^ While both silks show similar initial elastic moduli, their responses to strain differ significantly. Spider silk demonstrates a hardening trend upon uniaxial stretching, likely due to the alignment and extension of its protein chains and the reinforcement of its β-sheet nanocrystals. Silkworm silk, however, shows minimal change in elastic properties with tensile strain, which could be attributed to its less organized nanostructure and the potential fragmentation of its β-sheet crystallites under stress. These differences originate from their unique protein compositions and supramolecular assemblies. Spider silk, composed of major ampullate spidroins (MaSp1 and MaSp2) with highly aligned β-sheet nanocrystals, exhibits significant mechanical anisotropy and strain-hardening behavior.^79,80^ In contrast, *Bombyx mori* silk, comprising a heavy chain, light chain, and P25 glycoprotein, forms a semialigned hierarchical structure with moderate anisotropy and weak strain-dependence.^81−83^ The superior tensile strength, extensibility, and toughness of spider silk can be attributed to its higher proportion of proline residues, presence of tyrosine facilitating π-π stacking interactions, and a more organized nanostructure with interconnected β-sheet nanocrystals.

## 4 Conclusions

We have obtained the full elastic tensor and complete mechanical properties of the *Bombyx mori* silkworm silk in the natural and stretched states by using BLS experiments. The anisotropic mechanical properties of the silkworm silk stem from the underlying hierarchical structure, which disappears in the isotropic silk fibroin thin film. Notably, the axial and lateral Young’s moduli of the silkworm silk in the natural state are 23.4 ± 1.0 and 10.4 ± 0.5 GPa, respectively, leading to an elastic anisotropy of 2.3, similar to that of the spider silk. The mechanical properties of the silkworm silk exhibit a weak strain-dependence up to the breakage strain (∼20%), contrary to the strain-hardening behavior of the spider silk. The noncontact and noninvasive nature of BLS makes it a versatile experimental tool for understanding the comprehensive mechanical properties of structured materials. The complete elastic constants and mechanical properties of the silkworm silk and spider silk as well as their strain-dependences provide valuable data, which, together with future molecular dynamics simulations, could allow for further elucidation of the hierarchical structures of these two natural fibers of important applications and even development of high-performance synthetical fibers.

Our results indicate the importance of considering the full elastic tensor when characterizing biomaterials. The isotropic elasticity observed in regenerated silk fibroin films contrasts sharply with the anisotropic behavior of native silk fibers, indicating the critical role of protein assembly and orientation in determining mechanical properties. This difference can be ascribed to the different hierarchical structures of spider and silkworm silks. Spider dragline silk, composed of MaSp1 and MaSp2 proteins, forms highly aligned β-sheet nanocrystals interconnected by a network of less-ordered domains rich in β-turns and 31-helices. This organized structure likely contributes to its pronounced strain-hardening behavior and higher shear moduli. In contrast, *Bombyx mori* silkworm silk, consisting of heavy and light chain fibroins and P25 glycoprotein, forms a less-organized architecture with β-sheet nanocrystals (2–5 nm) only partially aligned along the fiber axis and embedded in a semiamorphous matrix. This structural difference can explain the weaker strain-dependence and lower shear moduli of silkworm silk, despite its slightly higher elastic anisotropy in the natural state. These findings show how subtle variations in protein composition and supramolecular assembly can lead to significant differences in macroscopic mechanical properties, providing valuable insights for the design of bioinspired synthetic fibers.

## Supporting Information Available

Transmission (a) and reflection (b) scattering geometries; antistokes spectra at θ = 60° at different α’s; transversely isotropic elasticity model; Brillouin light spectroscopy (BLS) experiments; χ2 fitting; and uncertainty quantification (PDF)
