# Supplementary material for: Elasticity Anisotropy of Bombyx mori Silkworm Silk Fiber by Brillouin Light Spectroscopy
Source: Biomacromolecules. 2025 Apr 1;26(4):2479–86. doi: 10.1021/acs.biomac.4c01844 (PMC12004526; doi:10.1021/acs.biomac.4c01844)
Supplement: Supplementary file 1 — bm4c01844_si_001.pdf [file bm4c01844_si_001.pdf]

Elasticity anisotropy of Bombyx mori silkworm silk fiber by Brillouin light spectroscopy

*Alina Aluculesei[1], Yuanzhong Zhang[2], Shifeng Huang[2], Zuyuan Wang[3], Yu Cang[4], Younjin Min[2,5], George Fytas[1,6]**

[1]Institute of Electronic Structure and Laser, FORTH, N. Plastira 100, 70013 Heraklion, Greece

[2]Department of Chemical and Environmental Engineering, University of California, Riverside, CA 92521, USA

[3]School of Mechanical and Electrical Engineering, University of Electronic Science and Technology of China, Chengdu, Sichuan 611731, PR China

[4]School of Aerospace Engineering and Applied Mechanics, Tongji University, Zhangwu Road 100, Shanghai 200092, China

[5]Material Science and Engineering Program, University of California, Riverside, CA 92521, USA

[6]Max Planck Institute for Polymer Research, Ackermannweg 10, 55128, Mainz, Germany

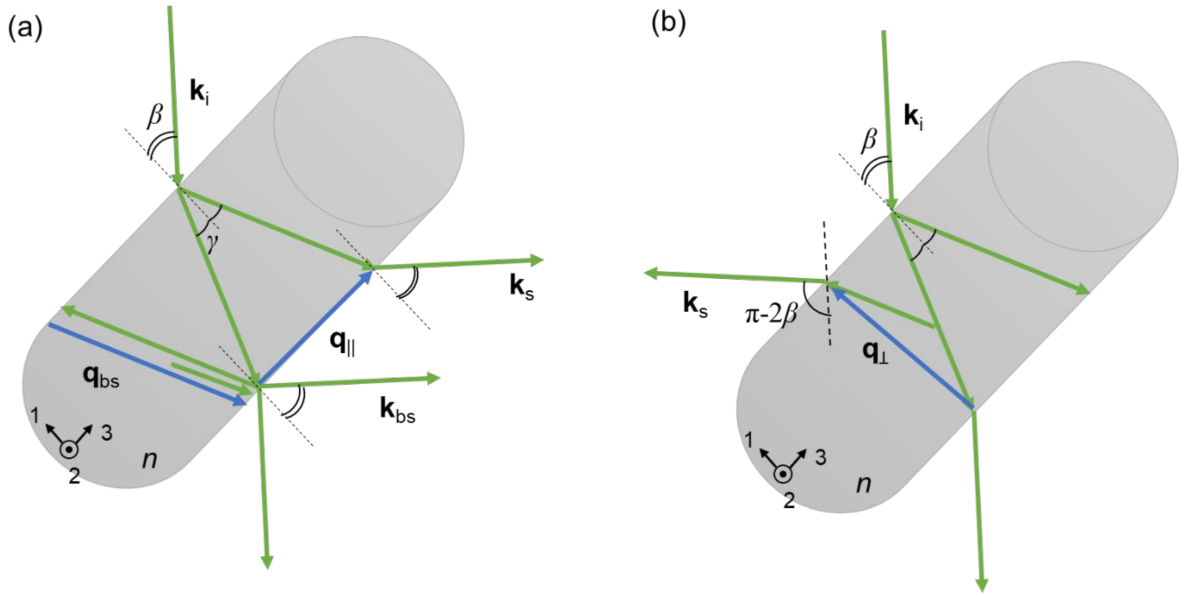

Figure S1. Transmission (a) and reflection (b) scattering geometries. In transmission geometry, we selected the scattered light $\mathbf{k}_s$ with the same incident angle β as the incident light $\mathbf{k}_i$. The corresponding $\mathbf{q}_{||}$ is therefore parallel to both the scattering plane formed by $\mathbf{k}_i$ and $\mathbf{k}_s$ and the principal direction of fiber. The scattered light includes components that are backscattered from reflections in the fiber, with the corresponding $\mathbf{q}_{BS}$ forming an oblique angle with the principal direction. In the reflection geometry, we selected the scattered light $\mathbf{k}_s$ such that its angle is complementary to the incident angle of the incident light $\mathbf{k}_i$, where the corresponding $\mathbf{q}_{\square}$ is normal to the scattering plane.

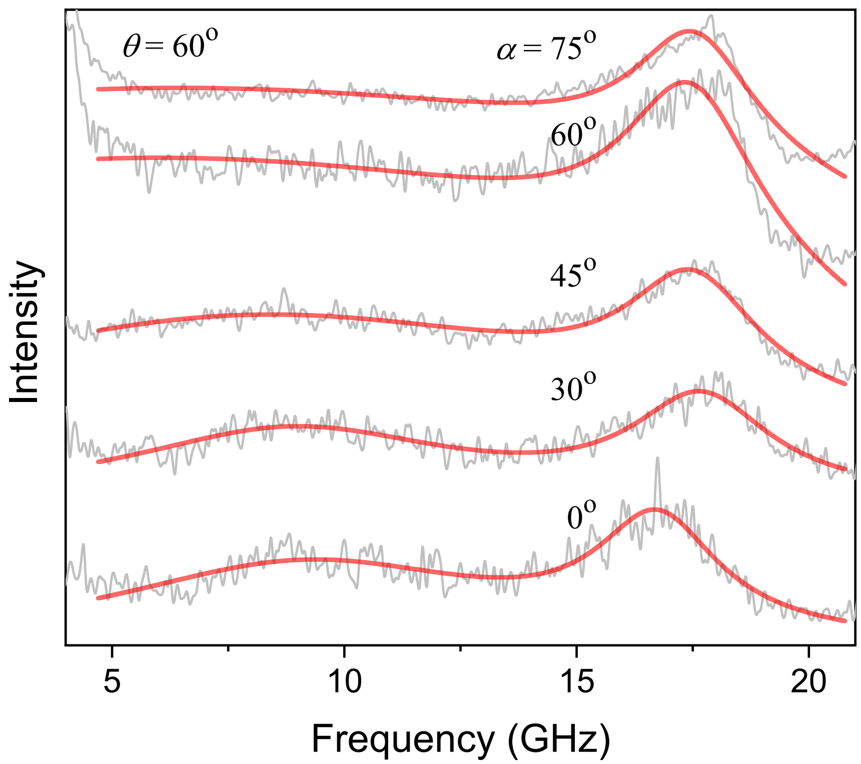

Figure S2. Anti-Stokes spectra at θ = 60° at different α's. The intensity of QL decreases as increasing α.

The intensity of scattered light is proportional to $\frac{|\xi^{\mu}|^2}{v_{\mu}^2}$, where ξ is the polarization vector of the scattered light by a phonon mode μ. Note μ=$L_{\|}$, $T_{\|,1}$, $T_{\|,2}$, $L_{\square}$, $T_{\square,1}$, $T_{\square,2}$, Q-L, Q-T, or P-T. The ξ expressions are useful for analysing the appearance. In transmission geometry, for $L_{\|}$ mode, $\xi_{VV}^{L_P}$ $= [0, -P_{13}, 0]$ which is detectable in VV and HH, but not in VH or HV. While in the backscattering geometry, for Q-L mode, $\xi_{VV}^{Q-L} = \left[0, -(P_{12}\sin^2\alpha + P_{13}\cos^2\alpha), 0\right]$ which is detectable in VV and HH, but not in VH or HV. In the spectra shown in Figure S2, the low- and high-frequency peaks could be assigned as $L_{//}$ and QL modes. The intensity of the $L_{//}$ mode is proportional to P_{13}^2. The intensity of the QL mode is mainly determined by P_{12}, P_{13}, and α. As α

increases from 0° to 75°, v_{QL} decreases from around 4960 to 3190 m/s (Figure 4), and $v_{QL}^2/v_{L//}^2$ decreases from 1 to around 0.41. Since $I_{L//}/I_{QL}$ decreases by more than 60% from α = 0° to 75° (Figure S2), it can be inferred that P_{12} should be larger than P_{13}.

Section S2. Transversely isotropic elasticity model

The silkworm silk was assumed to be transversely isotropic. To facilitate the analysis, a "123" coordinate system was constructed, with the "3"-axis parallel to the silk axis. For a transversely isotropic material, the elastic stiffness tensor contains 5 independent elastic constants (here chosen as C_{11}, C_{13}, C_{33}, C_{44}, C_{66}), and has the following form (in the Voigt notation),

$$\mathbf{C} = \begin{bmatrix} C_{11} & C_{11}-2C_{66} & C_{13} & & & \\ C_{11}-2C_{66} & C_{11} & C_{13} & & & \\ C_{13} & C_{13} & C_{33} & & & \\ & & & C_{44} & & \\ & & & & C_{44} & \\ & & & & & C_{66} \end{bmatrix}. \quad \text{(S1)}$$

The elastic stiffness constants are coupled with the direction-dependent sound velocities in the framework of the Christoffel's equation.[1, 2] Given the sound velocities, the elastic stiffness constants could be uniquely determined through least squares fitting. Because of the transverse isotropy, it is only necessary to consider phonon propagation in a quarter of the "23" plane, i.e., 0° ≤ α ≤ 90°. For a particular direction represented by an angle, α, there exist one quasi-longitudinal (Q-L) mode, one quasi-transverse (Q-T) mode, and one pure-transverse (P-T) mode. The direction-dependent sound velocities of the Q-L, Q-T, and P-T modes can be expressed as follows.

$$v_{\text{Q-L}}(\alpha) = \sqrt{\frac{-A_1 + \sqrt{A_1^2 - 4A_2}}{2\rho}} \quad \text{(S2)}$$

$$v_{\text{Q-T}}(\alpha)=\sqrt{\frac{-A_1-\sqrt{A_1^2-4A_2}}{2\rho}} \tag{S3}$$

$$v_{\text{P-T}}(\alpha)=\sqrt{\frac{A_3}{\rho}} \tag{S4}$$

where,

$$A_1=-(\sin^2\alpha C_{11}+\cos^2\alpha C_{33}+C_{44}) \tag{S5}$$

$$A_2=\sin^4\alpha C_{11}C_{44}+\sin^2\alpha\cos^2\alpha(C_{11}C_{33}-C_{13}^2-2C_{13}C_{44})+\cos^4\alpha C_{33}C_{44} \tag{S6}$$

$$A_3=\sin^2\alpha C_{66}+\cos^2\alpha C_{44}\,. \tag{S7}$$

For simplicity, the Q-L(0°), Q-T(0°), P-T(0°), Q-L(90°), Q-T(90°), P-T(90°), Q-L(0° < α < 90°), Q-T(0° < α < 90°), and P-T(0° < α < 90°) phonon modes are denoted as $L_{\|}$, $T_{\|,1}$, $T_{\|,2}$, $L_{\perp}$, $T_{\perp,1}$, $T_{\perp,2}$, Q-L, Q-T, and P-T, respectively. Since the PT mode is nondetectable except at α = 0°, the assumption of C_{66} = C_{44} is made [Czibula2024], further reducing the number of independent elastic constants to be four (i.e., C_{11}, C_{33}, C_{44}, C_{13}). The density ρ of the silkworm silk was assumed to be 1350 kg m^{-3}.[3]

Section S3. Brillouin light spectroscopy (BLS) experiments

The direction-dependent sound velocities were obtained by BLS, a non-contact, non-destructive technique. The experiments were conducted with a BLS setup, which includes a six-pass tandem Fabry-Perot interferometer and a Nd/YAG laser (λ_0 = 532 nm in air) mounted on a goniometer. The laser input power is around 5 mW. Transmission and reflection geometries were employed to probe phonons propagating in directions parallel, normal, and oblique to the silkworm silk axis. In the transmission and reflection geometries, the direction of the phonon wave vector remains unchanged, but its magnitude varies depending on the scattering angle. For the "artificial" backscattering modes observed in the transmission geometry, the magnitude of the phonon wave vector remains constant, but its direction varies depending on the laser incident angle. The polarization of the probed phonons (i.e., QL, QT, PT) was selected by using different polarization combinations of the incident and scattered light beams (e.g., VV, VH) and meanwhile taking into account the intensity of the scattered light. The magnitudes of the phonon wave vectors probed in the three scattering geometries (e.g., transmission, reflection, backscattering) with the different light polarization configurations (e.g., VV, VH) were calculated as follows.

In the transmission geometry:

$$q_{\parallel,\ \mathrm{VV/VH}} = q_{\parallel,\ \mathrm{HH}} = \frac{4\pi}{\lambda_0}\sin\beta \tag{S8}$$

In the reflection geometry:

$$q_{\perp,\ \mathrm{VV/VH}} = \frac{4\pi}{\lambda_0}\sqrt{n^2 - \sin^2\beta} \tag{S9}$$

In the "artificial" backscattering geometry:

$$q_{\mathrm{bs},\ \mathrm{VV/VH}} = \frac{4\pi n}{\lambda_0} \tag{S10}$$

Here, β is the laser incident angle, and n is the refractive index of the silkworm silk.

The sound velocity was calculated as,

$$v = \frac{2\pi f}{q}, \tag{S11}$$

where f is the phonon frequency obtained from the Lorentzian fits to the Brillouin peaks. In the experiments studying the strain effect on the silkworm silk's elastic properties, the silk fiber was stretched using a customized stretch meter by up to around 20%.

Section S5. χ^2 fitting

Based on the BLS-measured, direction-dependent sound velocities (i.e., $v_{Q\text{-}L}(\alpha)$, $v_{Q\text{-}T}(\alpha)$, $v_{P\text{-}T}(\alpha)$), nonlinear χ^2 fitting was conducted to obtain the elastic stiffness constants.[4] The χ^2 is defined as

$$\chi^2 = \sum_i \frac{\left[v_{i,\,\mathrm{fit}}(C_{11},\ C_{13},\ C_{33},\ C_{44},\ C_{66},\ \alpha) - v_{i,\,\exp}(\alpha)\right]^2}{(\Delta v_{i,\,\exp})^2}, \tag{S12}$$

where $v_{\mathrm{i,\,fit}}$ and $v_{\mathrm{i,\,exp}}$ are the fitted and experimental sound velocities, respectively, $\Delta v_{\mathrm{i,\,exp}}$ is the uncertainty of the measured sound velocity, and the summation is over all experimental sound velocities. By considering the measurement uncertainties of the angles, refractive indices, phonon frequencies, and so on, the relative uncertainty of $(\Delta v_{\mathrm{i,\,exp}})/(v_{\mathrm{i,\,exp}})$ was estimated to be 2%. To ensure positive Young's and shear moduli, the χ^2 fitting was performed subject to the following constraints: (i) $C_{11} > |C_{12}|$, (ii) $C_{44} > 0$, and (iii) $C_{33}(C_{11} + C_{12}) > 2C_{13}^2$.[5] Note $C_{12} = C_{11} - 2C_{66}$.

The independent elastic stiffness constants were used to predict the theoretical $v_{QL}(\alpha)$, $v_{QT}(\alpha)$, and $v_{PT}(\alpha)$, according to Eqs. (S2)-(S4). Furthermore, they were used to calculate the characteristic mechanical properties[6], including the axial and lateral Young's moduli ($E_{\|}$, $E_{\perp}$), shear moduli ($G_{13} = G_{23}$, G_{12}), bulk modulus (K), and Poisson's ratios ($v_{31} = v_{32}$, v_{12}). Note that only five of the engineering mechanical properties are independent. Typically, $\{E_{\|}, E_{\perp}, G_{13}, G_{12}, v_{31}\}$ or $\{E_{\|}, E_{\perp}, G_{13}, v_{12}, v_{31}\}$ are chosen.

Section S6. Uncertainty quantification

By considering the measurement uncertainties of the angles, refractive indices, phonon frequencies, and so on, the relative uncertainty of the BLS-measured sound velocities, $(\Delta v_{\mathrm{i,\ exp}})/(v_{\mathrm{i,\ exp}})$, was estimated to be 2%. The uncertainties of the elastic stiffness constants were determined by constructing a matrix **G**.[4] To simplify the notations, C_{11}, C_{13}, C_{33}, C_{44}, and C_{66} were denoted as a_1, a_2, a_3, a_4, and a_5, respectively. **G** has elements of the following form,

$$G_{mn} = \sum_i \frac{1}{(\Delta v_{i,\ \mathrm{exp}})^2} \frac{\partial v_{i,\ \mathrm{fit}}}{\partial a_m} \frac{\partial v_{i,\ \mathrm{fit}}}{\partial a_n}, \tag{S13}$$

where m and n (= 1, 2, 3) are matrix indices, and the summation is over all experimental sound velocities. Mathematically, **G** dictates how variations in the elastic constants affect the fitted sound velocities. The partial derivatives were approximated by using a central finite difference scheme. For example, $\frac{\partial v_{i,\ \mathrm{fit}}}{\partial a_m} = \frac{v_{i,\ \mathrm{fit}}(a_m + \delta a_m) - v_{i,\ \mathrm{fit}}(a_m - \delta a_m)}{2\delta a_m}$, where δa_{m} represents the change in a_{m}. From a convergence study, δa_{m} was determined to be $10^{-6} a_{\mathrm{m}}$. The inverse of **G** was calculated to obtain a covariance matrix, i.e., $\mathbf{M} = \mathbf{G}^{-1}$. The uncertainty of a_{m} is then the square root of the mth diagonal term of **M** (i.e., $\Delta a_m = \sqrt{M_{mm}}$).

The uncertainties of the Young's moduli, shear moduli, bulk modulus, and Poisson's ratios were calculated according to the principles of uncertainty propagation (i.e., the chain rule). For example,

$$\Delta E_{\mathrm{P}} = \sqrt{\sum_{ij=11,\ 13,\ 33,\ 66} \left(\frac{\partial E_{\mathrm{P}}}{\partial C_{ij}} \right)^2 \left(\Delta C_{ij} \right)^2 + \sum_{\substack{ij,\ kl=11,\ 13,\ 33,\ 66 \\ ij<kl}} 2 \frac{\partial E_{\mathrm{P}}}{\partial C_{ij}} \frac{\partial E_{\mathrm{P}}}{\partial C_{kl}} \left(\Delta C_{ij} \right) \left(\Delta C_{kl} \right)}, \tag{S17}$$

where $\frac{\partial E_{\mathrm{P}}}{\partial C_{11}} = \frac{C_{13}^2}{(C_{11}-C_{66})^2}$, $\frac{\partial E_{\mathrm{P}}}{\partial C_{13}} = -\frac{2C_{13}}{C_{11}-C_{66}}$, $\frac{\partial E_{\mathrm{P}}}{\partial C_{33}} = 1$, and $\frac{\partial E_{\mathrm{P}}}{\partial C_{66}} = -\frac{C_{13}^2}{(C_{11}-C_{66})^2}$. Here, $(\Delta C_{ij})(\Delta C_{kl})$ represents the covariance of C_{ij} and C_{kl}. $(\Delta C_{ij})(\Delta C_{kl})$ are the off-diagonal terms of the matrix **M**. For example, $(\Delta C_{11})(\Delta C_{13})$ = **M**(1, 2), $(\Delta C_{11})(\Delta C_{33})$ = **M**(1, 3), $(\Delta C_{11})(\Delta C_{66})$ = **M**(1, 5), $(\Delta C_{13})(\Delta C_{33})$ = **M**(2, 3), $(\Delta C_{13})(\Delta C_{66})$ = **M**(2, 5), and $(\Delta C_{33})(\Delta C_{66})$ = **M**(3, 5). Similarly, the expressions for $E_{\perp}$, G_{13}, G_{12}, K, v_{31}, and v_{12} could be derived.

References

(1) Cusack, S.; Lees, S. Variation of longitudinal acoustic velocity at gigahertz frequencies with water content in rat-tail tendon fibers. *Biopolymers: Original Research on Biomolecules* **1984**, *23* (2), 337-351.

(2) Wang, Z.; Cang, Y.; Kremer, F.; Thomas, E. L.; Fytas, G. Determination of the complete elasticity of Nephila pilipes spider silk. *Biomacromolecules* **2020**, *21* (3), 1179-1185.

(3) Freddi, G.; Romanò, M.; Massafra, M. R.; Tsukada, M. Silk fibroin/cellulose blend films: preparation, structure, and physical properties. *Journal of Applied Polymer Science* **1995**, *56* (12), 1537-1545.

(4) Zgonik, M.; Bernasconi, P.; Duelli, M.; Schlesser, R.; Günter, P.; Garrett, M.; Rytz, D.; Zhu, Y.; Wu, X. Dielectric, elastic, piezoelectric, electro-optic, and elasto-optic tensors of BaTiO 3 crystals. *Physical review B* **1994**, *50* (9), 5941.

(5) Mouhat, F.; Coudert, F.-X. Necessary and sufficient elastic stability conditions in various crystal systems. *Physical review B* **2014**, *90* (22), 224104.

(6) Cusack, S.; Miller, A. Determination of the elastic constants of collagen by Brillouin light scattering. *Journal of molecular biology* **1979**, *135* (1), 39-51.
